# The Effect of Contact Non-equilibrium Plasma on Structural and Magnetic Properties of Mn_*Х*_Fe_3 − *X*_О_4_ Spinels

**DOI:** 10.1186/s11671-017-2268-5

**Published:** 2017-08-23

**Authors:** L.A. Frolova, M. P. Derhachov

**Affiliations:** 1 Ukrainian State University of Chemical Technology, Gagarin Ave., 8, Dnipropetrovsk, 49005 Ukraine; 2 Oles Honchar Dnipropetrovsk National University, Gagarin Ave., 72, Dnipropetrovsk, 49010 Ukraine

**Keywords:** MnFe_2_O_4_ spinel, Preparation, Combustion, Chemical precipitation, Characterization, CNP

## Abstract

Nano-sized manganese ferrites Mn_*х*_Fe_3 − *х*_О_4_ (*х* = 0–1.3) were prepared using contact non-equilibrium plasma (CNP) in two different pH (11.5 and 12.5). The influence of synthesis conditions (e.g., cation ratio and initial pH) on phase composition, crystallite size, and magnetic properties were investigated employing X-ray diffraction (XRD), differential thermal analysis (DTA), Fourier transform infrared (FTIR), scanning electron microscopy (SEM), transmission electron microscopy (TEM), and magnetic measurement techniques. The formation of monodispersed faceted ferrite particles at *х* = 0–0.8 was shown. The FTIR spectra revealed reflection in region 1200–1700 cm^−1^ caused by the presence of water adsorbed on the surface of Fe_3 − *x*_Mn_*x*_O_4_ micro-granules or embedded into their crystal lattice. The most sensitivity of reflection spectra to the composition changes takes place within a 400–1200 cm^−1^ range, typical to the stretching vibrations of Fe(Mn)–O (up to 700 cm^−1^ ), Fe(Mn)–OH, and Fe(Mn)–OH_2_ bonds (over 700 cm^−1^). The XRD results showed that the nanocrystalline Mn_*х*_Fe_3 − *х*_О_4_ (0 < *x* < 1.0) had cubic spinel crystal structure with average crystallite size 48–49 A. The decrease of crystalline size with the *x* increase was also observed.

## Background

The ability of nanodispersive spinels with polyvalent metals to form a number of solid solutions and compounds gives unlimited possibilities to control technological properties of spinel compounds. For a long time, great attention of many researchers has been paid to the investigations of manganese ferrites (Fe_3_O_4_ − Mn_3_O_4_ system) because of their wide application in industry. They are widely used in microwave ovens and magnetic storage devices, as well as highly active catalysts in producing hydrogen via methane dehydrogenation into ethylene or acetylene, adsorbents [1–6].

The synthesis of manganese ferrite spinel is technologically complex. Presently, there are few methods for the synthesis of manganese ferrite particles, such as ceramic [7], coprecipitation [8–12], hydrothermal method [13], reverse micelle [14, 15], sol-gel [16], combustion method [17], mechanosynthesis [18–20], high-energy technologies [21, 22], and mechanical doping [23, 24]. Hydrophase methods allow regulating composition, crystallinity, and particle morphology.

Such methods have been studied by many researchers and are successfully applied for synthesis of ferrites [9, 25, 26] with particle size of 30–50 nm at 50–150 °С, which is significantly lower than for ceramic technology. Hydrophase methods, as a rule, include several stages: the first—deposition, the second—directly ferrite synthesis, carried out due to oxidation, aging, etc. The methods for initiation of the second main stage of ferrite synthesis using ultrasound treatment, microwave influence, ultraviolet, and various discharges [27–29] have been used recently. During the treatment of solutions with discharge of CNP, a complicated complex of chemical reactions involving radical particles and free electrons occurs. The main products of such interactions are oxygen, hydrogen, and hydrogen peroxide. Oxidative activity of plasmochemically “activated” solutions can be used for synthesis of complex oxide compounds.

The emission spectrum [30–32] has shown that the main contributions to the emission spectrum of the water vapor plasma are OH, atomic hydrogen, and the oxygen radical. In the case of the bubble mode, when the streamers fill the entire bubble, a significant emission from the nitrogen second positive system and the nitrogen ion (first negative system). The discharge operates in two different modes. For small conductivities of the liquid, the discharge is a direct liquid streamer discharge (liquid mode). This mode is similar to the typical so-called corona discharges in water. For conductivities above typically 45 μS cm^−1^ a large vapor bubble is formed. In the bubble mode, the streamers are located at the bubble–liquid interface. The hydrogen peroxide formation efficiency is dependent on power with a maximum for intermediate powers. The hydrogen peroxide formation efficiency is significantly smaller in the bubble mode than in the liquid mode. In the work [33], the kinetic parameters of electrons for the dielectric barrier discharge with a liquid electrode at atmospheric pressure have been estimated. Thus, we may suppose that the CNP will possess chemical activity with respect to its application in realization of the different oxidative-reductive processes.

Our preliminary studies of plasma treatment of solutions have shown that the composition of synthesized oxidizer solution depends on a wide range of factors [29]. The use of CNP guarantees high degree of homogeneity in component distribution both in the initial solution and in the product formed during oxidation, which stimulates effective interaction between them with formation of ferrites with homogeneous structure and composition.

The aim of the work is to study the possibility of obtaining nano-sized Mn_*Х*_Fe_3 − *Х*_О_4_ spinel from aqueous solutions using contact non-equilibrium plasma. Since ferrites are solid solutions, it is important to establish the degree of their structural and concentration homogeneity under the selected synthesis conditions. The experimental method consisted in comparison of ferrospinel obtained from manganese and iron sulfates at different cation ratio.

Such comparative research of the samples allow establishing the influence of the chemical composition of the initial solution and the synthesis conditions on the structural-phase state of the compounds prepared using CNP treatment.

## Methods

For the synthesis of manganese ferrite, the authors have used aqueous solutions of FeSO_4_·7H_2_O, MnSO_4_·5H_2_O, and aqueous solution of NaOH was used as a precipitant. We used 0.5 M solutions of iron and manganese salts. All the chemicals and solvents employed for the synthesis were of analytical grade and used as received without further purification. Deionized water was used as solvent in whole procedure.

Preliminary studies [25] showed that at pH < 11 non-magnetic oxides and oxyhydroxides were formed, so two sets of samples were prepared. The first set at initial рН = 11.5 and the second at 12.5. The coprecipitated compounds were prepared by pouring at continuous stirring of corresponding mixture of sulfate solutions with necessary cation ratio. The further treatment was carried out using CNP.

The treatment was conducted in a cylindrical reactor with inner diameter of 45 mm and height of 85 mm. The reaction mixture was cooled by the continuous circulation of cold water in the outer jacket. One of stainless steel electrodes (diameter 4 mm) was located in the lower part of the reactor, and the other (diameter 2.4 mm) was located 10 mm above the surface of the solution. The initial voltage was delivered to the step-up transformer. The ac current from the secondary coil was delivered to the bridge rectifier and then, now pulsating voltage, was delivered via a ballast resistor to the reactor electrodes. The igniting unit was additionally connected to the anode. This unit formed pulses with amplitude of up to 15 kV at a width of 1.5 ms. The pulses were strictly synchronized with the phase of the pulsating voltage. At the instant when the igniting pulse was formed, there was a breakdown between the reactor electrodes in the vacuum space created by rarefaction to 0.06–0.08 MPa. The resistance sharply dropped, and an anode current started to flow thereby creating a discharge. The discharge burning voltage remained nearly unchanged at 750–900 V. The current in the discharge gap was determined by the plasma resistance and the voltage applied to the system formed by the plasma discharge and the ballast controller. The voltage was controlled by the phase method principle, i.e., the average anode voltage applied to the reactor depended on the phase of the pulsating voltage at the anode and on the instant at which an ignition pulse was delivered.

**Fig. 1:**
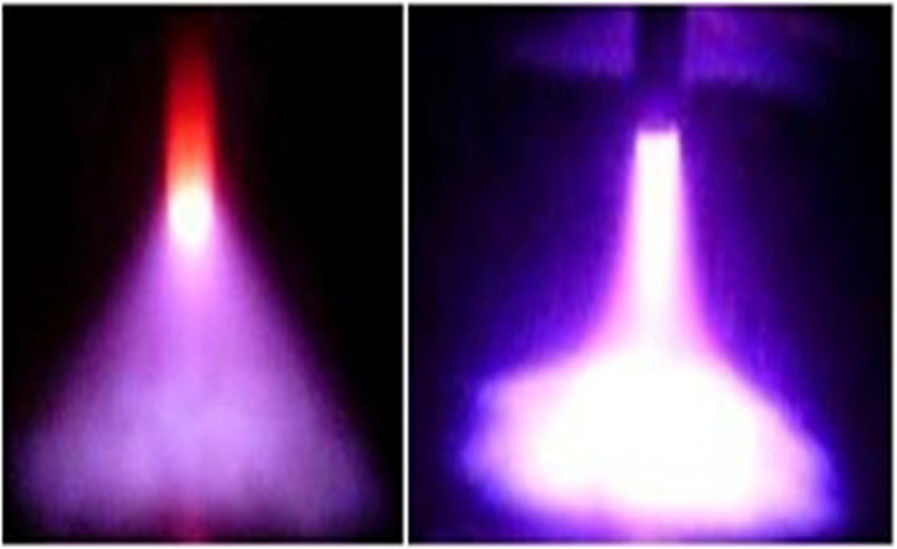
The pillar of contact non-equilibrium plasma between the electrode in the gas phase and the surface of the liquid

The plasma appeared at the ignition instant and was extinguished when the anode voltage pulsations terminated (Fig. 1). The repetition frequency of the process was 100 Hz. The discharge current was controlled by changing the instant of ignition relative to the phase of anode voltage pulsations with a synchronizing device. The duration of plasma treatment varied from 10 to 40 min. All precipitates were washed until negative reaction on sulfate-ion. The washed and filtered precipitates were dried at 150 °С. Relative magnetic properties (of saturation magnetization *I*_S_ (emu^2^/g), coercive force Нс (Oe)) were evaluated by magnetometer [29].

The concentration of Mn^2+^ in the samples obtained was determined complexometrically. The concentration of iron was determined using permanganate and bichromate methods. In order to monitor the reaction process, the reactor was equipped with an electrode system. The [Fe^2+^]/[Mn^2+^] ratio in Mn_*х*_Fe_3 − *х*_О_4_ compound was calculated according to formula:

$$ \frac{C_{\mathrm{Mn}}}{C_{\mathrm{Fe}}}=\frac{x}{3-x} $$

and values were equal of *х* = 0; 0.2, 0.4, 0.6, 0.8, 0.9, 1, 1.1, 1.2, and 1.3 were chosen. Fourier transform infrared reflection spectra of manganese ferrites Mn_*x*_Fe_3 − *x*_O_4_ (*x* = 0.0, 0.2, 0.4, 0.6, 0.8, 0.9, 1.0, 1.1, 1.2, 1.3) were measured within a 400–4000 cm^−1^ range by employing a Fourier transform infrared (FTIR) spectrometer Nicolet iS10. To study the transformations occurring upon heating of obtained powders, we used differential thermal analysis (DTA) and differential thermogravimetric analysis (DTG). DTA, mass loss TG, and mass loss rate DTG curves were recorded on Derivatograph Q-1500D (F. Paulik, J. Paulik, and L. Erdey). The temperature was varied in range 20–1000 °C at heating rate of 10°/min. γ-Al_2_O_3_ was used as a reference. The mass of each sample was 200 mg. The morphology of the ferrite powders and the particle size were studied using scanning electron microscopy. The phase composition (XRD) and the structure of the ferrite samples were studied using X-ray diffractometer DRON-2 in monochromatized Cо-K_α_ radiation. The crystallite size and the degree of microstrains were calculated using approximation method. The size and the shape of the particles were determined using electron Microscope “Jem 1010” (JEOL) at working value voltage of 200 kV. Scanning electron microscopy with X-ray microanalysis was carried out using REMMA-102 (SELMI, Ukraine).

## Results and Discussion

Properties of magnetic materials based on manganese ferrites depend on their structural and phase state. The synthesis of such ferrites call for preparation of single-phase product with spinel structure not having residual iron oxide or other phases, which are intermediate products of ferrite formation from oxides. On par with phase composition, the magnetic properties are significantly influenced by oxidation of iron and manganese cations, and character of their location in site of spinel crystal lattice. It is known that divalent cations (Zn^2+^, Mn^2+^) are mostly located in tetrahedral positions and trivalent (Fe^3+^)—in octahedral positions of spinel crystal lattice. According to Néel relaxation theory, such arrangement provides maximum value of material magnetization. During preparation of ferrites, oxidation of Mn^2+^ to Mn^3+^ is possible, which can be accompanied by reduction of Fe^3+^ to Fe^2+^ and rearrangement of cations in sublattices, with partial transfer of Fe^2+^ into tetrahedral and Mn^3+^—into octahedral nodes of crystal lattice, which negatively impacts magnetic properties of ferrites. Oxidation of Mn^2+^ occurs at highest rate at 900–1000 °С, and optimal condition for sintering of manganese ferrites for ceramic technology—1000–1200 °С. The data available in various literature sources on discussion of magnetic structure and properties of manganese ferrites are contradictory, which is likely related to variation in arrangement of iron and manganese ions and their polyvalency. The data of the dependence of lattice parameter on the value of *х* in case of various technologies make it possible to assume the character of cation arrangement in the lattice.

**Fig. 2:**
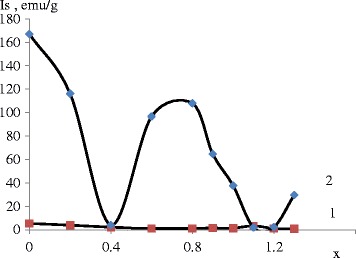
Dependence of saturation magnetization on cation molar ratio at different рН: 1—рН = 11.5 and 2—рН = 12.5

**Fig. 3:**
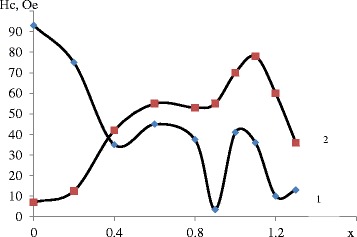
Dependence of coercive force on cation molar ration at different рН: 1—рН = 11.5 and 2—рН = 12.5

The assessment results of prepared samples can be formulated as follows: all samples include chemically bound water in various amounts. In both sets, the highest water content is found in samples with *х* = 0.4, 1.1…1.3. The first set showed weak magnetic properties (Figs. 2 and 3), and so it was not considered in detail.

As can be seen from Fig. 1, there are some differences in saturation magnetization of both sets. The highest value for set 1 corresponds to ratio 1,1 Mn_1.0_Fe_0.9_Mn_0.1_О_4_. The highest value is achieved at рН = 12.5 and ratio of *х* = 0.8 (Mn_0.8_Fe_0.2_Fe_2_О_4_). This ratio is different from stoichiometric manganese ferrite.

By assessing saturation magnetization, it can be said that sample nos. 1, 2, 3, and 8 have lower values due to their amorphous structure and presence of non-magnetic phases.

**Fig. 4:**
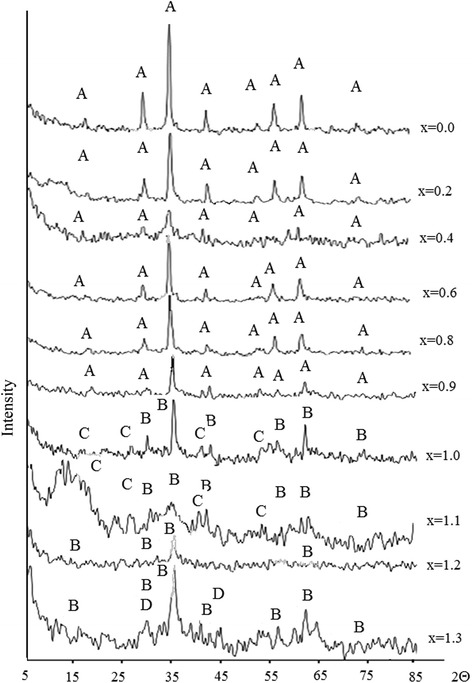
XRD patterns of ferrite obtained at different ratios of components (Table 1): A—Fe_3_O_4_, B—MnFe_2_O_4_, C—Mn_3_O_4_, and D—β-MnO_2_

Figure 4 shows XRD patterns of samples from the second set. The XRD patterns may be divided into two categories—first samples 6–10 that have monophase crystal structure corresponding to spinel phase ferrite (JCPDS 10-0467). Relatively sharp and intense lines of spinel phase ferrites can be observed on XRD patterns of the samples. The lines related to oxide phases of Fe_2_O_3_ and MnO_*x*_ are absent on XRD patterns (Fig. 4).

The second set has less crystalline with few phases present. On XRD patterns of the samples, prepared with higher manganese content, the lines are slightly broadened, which may indicate changes in its structure in comparison with the stoichiometric sample. Presence of other phases in case of higher manganese content was found using XRD method. The broad peaks can be observed on XRD patterns, which can be indexed as (311) of ferrite’s spinel phase (JCPDS 74-2403). In the region of angles corresponding to the highest intensity lines for Fe_3_O_4_ and Mn_3_O_4_, a halo of low intensity is observed, which can indicate the presence of these oxides in the samples. There is a clear correlation between the magnetic characteristics and the degree of crystallinity and homogeneity of the product.

**Fig. 5:**
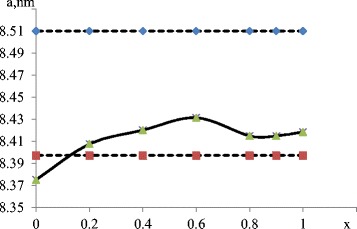
Dependence of crystal lattice parameter on cation ratio *х*

Taking into account that Mn^2+^ cations are the largest of all, it could be assumed that as the value of *x* increases, it is possible to increase the lattice parameter. The analysis of XRD patterns (Fig. 5) shows that the parameter of crystal lattice *а* = 8.4196 А (for stoichiometric tetragonal manganese ferrite MnFe_2_O_4_ *а* = 8.51 А). Noticeably smaller value of lattice parameter can be explained with formation of manganese ferrite at рН = 12.5 following magnetite formation mechanism. Upon oxidation of Mn^2+^:

3Mn^2+^→2Mn^3+^+□

vacancies are formed, which facilitate reduction of lattice parameters. Magnetite is formed in the second set, and gradual substitution of iron cations with manganese cations leads to reduction of magnetic properties to ratio 0.4 (the first peak) following 1-1,1, corresponding to stoichiometric manganese ferrite. Analysis of Figs. 2 and 3 makes it possible to establish that the formation of compounds in the second set occurs according to the maghemite formation mechanism.

As stated in Table 1, ferrite Mn_*Х*_Fe_3 − *X*_О_4_ was obtained in the nanorange. The average crystallite size of nanoparticles Mn_*Х*_Fe_3 − *X*_О_4_ were ranged from 5 to 8 nm and reached a maximum at *x* = 0. The calculated Mn_*Х*_Fe_3 − *X*_О_4_ crystalline size exceeded ferrite crystallite size in TEM image in four times due to aggregation of nanoparticles.

**Table 1:** Dependence of the main characteristics of products on composition

Sample number	*х*	Composition	а, А	Crystallite size, А	T_C_, °С	Mass loss, %	Literature value а, А
1	1.3	Mn_1.3_Fe_1.7_О_4_	Amorphous		315	11.5	
2	1.2	Mn_1.2_Fe_1.8_О_4_	Amorphous		315	19.6	
3	1.1	Mn_1.1_Fe_1.9_О_4_	Amorphous		305	16.4	
4	1	MnFe_2_О_4_	8.4184	48.8	320	13.4	8.51
5	0.9	Mn_0.9_Fe_2.1_О_4_	8.4148	52.3	280	6.7	
6	0.8	Mn_0.8_Fe_2.2_О_4_	8.4148	60.9	315	7.2	
7	0.6	Mn_0.6_Fe_2.4_О_4_	8.4313	66.1	315	5.3	
8	0.4	Mn_0.4_Fe_2.6_О_4_	Amorphous		320	15.4	
9	0.2	Mn_0.2_Fe_2.8_О_4_	8.4075	66.3	580	6.2	
10	0	Fe_3_О_4_	8.3750	72.3	520	0	8.397

Also, Table 1 shows the variation of Curie temperature, lattice parameter with ratio *x* in FeMn_2 − *x*_O_4_. Curie temperature decreases as cations manganese content increases. As it can be known, Curie temperature is mainly determined by the strongest super exchange interaction in ferrites. The factors decreasing this interaction lead to a decrease in Curie temperature. With an increase in the manganese content, the lattice parameter increases (Table 1). This leads to an increase in ionic distances as well as decrease in Curie temperature.

**Fig. 6:**
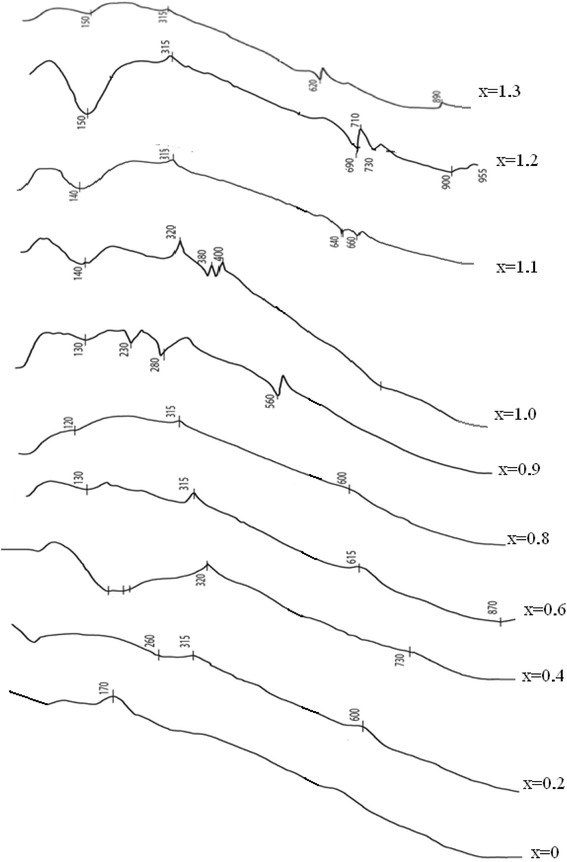
Derivatography patterns of samples synthesized at рН = 12.5

The present assumption requires additional study. Analysis of derivatography patterns indicates on formation of manganese ferrite in sample nos. 4 and 5 and isomorphism of properties for samples 5–10 (Fig. 6). The compounds of various composition are formed in samples 1–5. The lowest mass losses are also observed for stoichiometric compositions. The first regions of derivatography patterns demonstrate various endo- and exothermic effects corresponding to oxidation of manganese and iron cations. High temperature region corresponds to rearrangement of crystal lattice (endo-effects without changing the mass).

DTG curves demonstrate that for all compositions the main mass loss corresponds to the loss of free water at 100 °С and bound at 160 °С. For composition 4, corresponding to stoichiometric ferrite, exothermic peaks are observed, which correspond to oxidation of manganese cation to various oxidation states. In the work [34], the authors have presented the following set of reaction occurring at various temperatures.

1. 3 Fe^2+^→2Fe^3+^+□temperature 280 °C
2. 4 Mn^3+^→3Mn^4+^+□temperature 330 °C
3. 3 Mn^2+^→2Mn^3+^+□temperature 360 °C
4. 3Mn^4+^ +□→4Mn^3+^temperature 420 °C
5. 2Mn^2+^→Mn^3+^ temperature 600 °C

Upon heating to 450–500 °С a structure of γ-Fe_2_O_3_ type is formed.

It can be assumed, that peaks at 600 °С correspond to oxidation and reduction of iron and manganese cations. Further oxidation is accompanied with transition from cubic to rhombohedral lattice, in which all cations are trivalent. The formation of α-Fe_2_O_3_ and α-Mn_2_O_3_ occurs in range from 600 to 1000 °С. XRD analysis of products obtained after heating to 1000 °С indicates the presence of magnetic phase of rhombohedral manganese ferrite for the samples with stoichiometric ratio of iron and manganese formed from iron and manganese oxides.

In addition, upon heading samples 1–10 to 1000 °С (Table 1), the formation of complex iron and manganese oxide occurs via similar mechanism. The formed compounds have similar peaks regardless of the initial composition. This is related to rhombohedral structure, in which all cations are trivalent. Since hematite and hausmannite have similar structure, all XRD patterns have similar character.

**Fig. 7:**
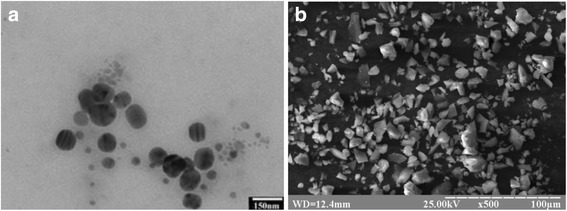
TEM image (**a**) and SEM image (**b**) of sample no. 4 set 2

According to TEM results, all samples synthesized using CNP method are composed of particle with regular faceted shape, with size ranging from 50 to 100 nm (Fig. 7). The product is monodisperse with average particle size 70–80 nm. The observed faceted particles are polycrystalline. Data acquired using SEM confirm that large ferrite particles are composed of very small primal particles and their size is not in agreement with values calculated using crystallite size (Table 1).

It is known from literature sources that in IR patterns of γ-Fe_2_O_3_ and Fe_3_O_4_, there are two main groups of characteristic lines allowing to judge intricate structural differences. These are lines related to vibrations of М–О and М–О–Н bonds. Introduction of different metal ions into iron oxide, causing symmetry distortion of coordination environment of Fe^3+^ or changes in Fe–O bond constant, can lead to the splitting or shifting of characteristic lines of Fe–O bond vibrations. In case of homogeneous distribution of ion of different nature in crystal lattice of spinel structure, we can usually observe only shift of absorption line’s maxima of characteristic oscillations.

**Fig. 8:**
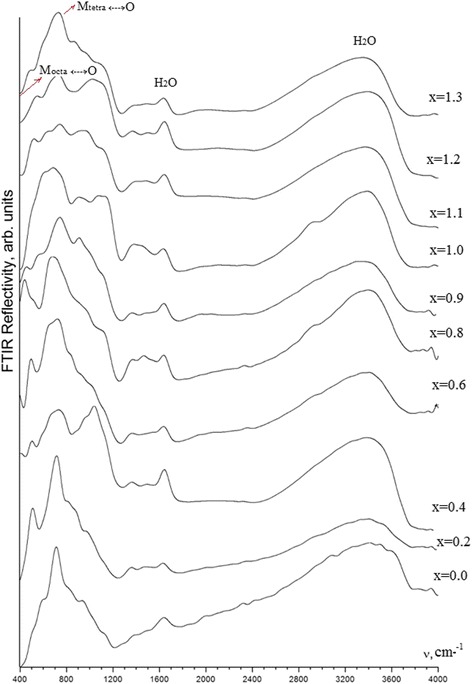
IR reflection spectra of samples with synthesized рН = 12.5 at different cation ratio

Figure 8 shows IR spectra of the studied samples. The spectral distribution over 1200 cm^−1^ is quite independent of the sample composition (Fig. 8).

Reflection in this region is caused by the presence of water that is adsorbed on the surface of Fe_3 − *x*_Mn_*x*_O_4_ micro-granules or embedded into their crystal lattice. The bands within a 1200–1700 cm^−1^ range are related to the bending H–O–H vibrations, and those within a 2400–3700 cm^−1^ range are due to the stretching vibrations of O–H bonds.

The most sensitivity of reflection spectra to the composition changes takes place within a 400–1200 cm^−1^ range, typical to the stretching vibrations of Fe(Mn)–O (up to 700 cm^−1^), Fe(Mn)–OH and Fe(Mn)–OH_2_ bonds (over 700 cm^−1^). The spectral position of the most intensive band is varied with the *x* changing. Its most shift, from 715 cm^−1^ in the Fe_3_O_4_ spectrum (*x* = 0.0) up to 688 cm^−1^, occurs for the sample with *x* = 0.8. The broadening of this band with the *x* increase is also observed (Fig. 8). Moreover, a new band at 445 cm^−1^ is confidently detected in the spectra of samples with *x* = 0.8 and 0.9. In addition to these features, we should mention a significant spectral redistribution in the *x* = 0.4 spectrum, as a result of the raising of 1039 cm^−1^ reflection band relatively to the band at 715 cm^−1^ in the *x* = 0.0 spectrum.

In accordance with crystallographic data, metal (Mn, Fe) ions may occupy positions with tetrahedral and octahedral oxygen neighboring [35]. The most probable positions for manganese ions at concentration of *x* < 1.3 are the tetrahedral positions corresponding to the Mn^2+^ charge state. The appearance of the octahedral-coordinated manganese ions with the same charge state is detected for the values of *x* within a 0.8–1.2 range. The filling of octahedral positions with Mn^3+^ ions starts at *x* = 1.0, and the part of them at *x* = 1.3 is no more than 23% from total quantity of manganese ions [35].

This is the reason to explain the changes observed in the *x* = 0.8 spectrum by starting the filling of octahedral positions with Mn^2+^ ions.

The raising of the 1039 cm^−1^ band in the *x* = 0.4 spectrum may be related to the structural variations in the metal (Mn, Fe) ions neighboring, that results in dipole momentum changing.

More detailed analysis is, unfortunately, complicated by essential overlapping of broadened bands that is typical for solid solutions containing tetrahedral and octahedral complexes with central atoms whose masses are close to each other.

## Conclusions

In the present work, we have found a new route for the synthesis of ultrafine manganese ferrite of type Mn_*Х*_Fe_3 − *X*_О_4_ in a wide Mn^2+^ substitution range from *x* by coprecipitation with CNP treatment. Coprecipitation followed by CNP treatment is an effective method for the preparation of manganese ferrite powder. The magnetic properties of Mn_*Х*_Fe_3 − *X*_О_4_ samples were increased with increasing pH values. Ferritization process was effective only at pH = 12.5. The formation of compounds at pH = 11.5 occurs by the mechanism of the formation of maghemite. High magnetic properties exhibited nanodispersed ferrite obtained at pH = 12.5, *x* = 0.6–0.8. The average crystallite size ranged from 50 to 80 A. The nanodispersed ferrites had a faceted shape and uniform particles. XRD pattern indicates the single spinel phase nanocrystals with cubic spinel structure at 0 < *x* <0.8.

FTIR spectroscopy confirmed the results of magnetic measurements. The decrease in the value of magnetic saturation beginning with *x* = 1.0 is due to the filling of octahedral positions with Mn^2+^ ions.

## Abbreviations

CNP: Contact non-equilibrium plasma
DTA: Differential thermal analysis
DTG: Differential thermogravimetric analysis
FTIR: Fourier transform infrared
I_S_: Saturation magnetization
SEM: Scanning electron microscopy
T_C_: Temperature
TG: Mass loss
XRD: X-ray diffraction
Нс: Coercive force (Oe)
